# Early-Stage Fault Diagnosis of Motor Bearing Based on Kurtosis Weighting and Fusion of Current–Vibration Signals

**DOI:** 10.3390/s24113373

**Published:** 2024-05-24

**Authors:** Bingye Zhang, Haibo Li, Weiyi Kong, Minjie Fu, Jien Ma

**Affiliations:** 1 State Grid Taizhou Power Company, Taizhou 318000, China; lihaibo202404@163.com (H.L.); kongweiyi202404@163.com (W.K.); 2 College of Electrical Engineering, Zhejiang University, Hangzhou 310007, China; 3190100953@zju.edu.cn (M.F.); jienma@126.com (J.M.)

**Keywords:** DLMD, fusion of current–vibration signals, kurtosis value, rolling bearing, fault diagnosis

## Abstract

To solve the problem of a low signal-to-noise ratio of fault signals and the difficulty in effectively and accurately identifying the fault state in the early stage of motor bearing fault occurrence, this paper proposes an early fault diagnosis method for bearings based on the Differential Local Mean Decomposition (DLMD) and fusion of current–vibration signals. This method uses DLMD to decompose the current signal and vibration signal, respectively, and weights the decomposed product function (PF) according to the kurtosis value to reconstruct the signal, and then fuses the reconstructed signals to obtain the current–vibration fusion signal after normalization, and then analyzes the fusion signal spectrally through the Hilbert envelope spectrum. Finally, the fusion signal is analyzed by the Hilbert envelope spectrum, and a clear fault characteristic frequency is obtained. The experimental results demonstrate that compared to traditional bearing fault diagnosis methods, the proposed method significantly improves the signal-to-noise ratio of fault signals, effectively enhances the sensitivity of early-stage fault detection in motor bearings, and improves the accuracy of fault identification.

## 1. Introduction

A permanent magnet synchronous motor (PMSM), known for its high operational efficiency and excellent power performance indices, is widely used in industrial robots, electric vehicles, CNC machine tools, and other industrial fields. As a crucial industrial energy conversion device, the health and reliable operation of a permanent magnet synchronous motor are vitally important to ensure the continuity of industrial production and to enhance the safety of transportation. Statistics show that about 41% of common motor faults are caused by bearing failures [1]. Therefore, fault diagnosis of bearings has always been a hot topic in the field of motor fault diagnosis.

Enhancing the sensitivity and accuracy of detecting faults, especially in their early stages, is a critical aspect of bearing fault diagnosis. Presently, vibration signal analysis techniques are widely utilized for this purpose [2,3]. In the case of motors, stator current signals carry valuable information about bearing faults, hence making Motor Current Signature Analysis (MCSA) a prevalent diagnostic method due to its affordability and simplicity in signal acquisition [4]. However, in the early stages of bearing issues, fault signal frequency amplitudes are minimal, and signal-to-noise ratios are low, posing challenges to accurately and effectively diagnose motor bearing conditions using conventional vibration and stator current signal analysis methods due to their limited sensitivity and susceptibility to noise harmonics.

To resolve the issue of weak fault characteristic signals and significant noise interference during bearing fault diagnosis, signal decomposition methods are widely used for fault signal denoising. The Empirical Mode Decomposition (EMD), proposed by Huang et al. [5], adaptively decomposes signals into IMF components, but this method suffers from mode mixing [6]. Smith et al. [7] proposed Local Mean Decomposition (LMD), which somewhat mitigates mode mixing and endpoint effects but still faces issues like the appearance of spurious frequencies [8], making it unsuitable for identifying and extracting weak signals in the early stages of bearing faults. Based on this, the Differential Local Mean Decomposition (DLMD) [9] was proposed, incorporating differential and integral operations during the LMD process, which enhances the amplitude ratio between frequencies and effectively suppresses the generation of spurious interference frequencies, making it suitable for early-stage bearing fault diagnosis.

To further highlight the fault characteristic frequencies in the early stages of bearing faults and to suppress noise interference in diagnostic accuracy, many researchers employ signal-weighted fusion methods to process fault signals. Wang [10] proposed a multimodal sensor fusion method that integrates vibration and sound signals to extract fault characteristics, enhancing diagnostic accuracy. Lu et al. [11] introduced a fault detection method weighted by a grey relational degree, calculating weight ratios through the entropy weighting method for weighted fusion. Bai et al. [12] proposed a fault detection method weighted by a B-type relational degree, weighting the IMF components according to the B-type relational degree to improve diagnostic accuracy.

Based on the analysis above, to address the issues of low sensitivity and susceptibility to noise harmonic interference in the early-stage motor bearing fault diagnosis, this paper proposes a bearing fault diagnosis method based on DLMD and the fusion of current–vibration signals. This method uses DLMD to decompose both current and vibration signals separately, reconstructs the signals weighted according to the kurtosis values of the decomposed PFs, normalizes the reconstructed signals, and fuses them to obtain a current–vibration fusion signal. The fusion signal is then spectrally analyzed using the Hilbert envelope spectrum to obtain clear fault characteristic frequencies. By employing DLMD and signal-weighted fusion, the signal-to-noise ratio of the fault characteristic signal is significantly improved, effectively suppressing mode mixing, and thereby enhancing the sensitivity and accuracy of early-stage bearing fault detection.

## 2. Fault Signal Analysis Fundamentals

### 2.1. Analysis of Fault Characteristics in Current Signals

Most bearings used in PMSM are rolling bearings, consisting of the outer ring, inner ring, rolling elements, and cage.

Bearing faults manifest as wear, fretting, cracking, brinelling, and scuffing, among others. Bearing damage can be categorized into localized damage and wear-related failures based on the vibrations they produce [13]. Wear-related failures such as wear, fretting, and brinelling are gradual faults. Typically, in the early stages of these faults, they do not immediately affect the operation of the motor, and their harmfulness is much less than that of localized damage failures. Moreover, they often exhibit strong randomness and irregular vibrations in vibration analysis, making it challenging to diagnose wear-related faults through signal analysis. Therefore, when conducting research on bearing fault diagnosis, wear-related faults are generally not considered. Localized bearing faults include fatigue spalling, local pitting, and cracks, among others. Depending on the location of occurrence, they typically manifest in three types: outer ring faults, inner ring faults, and rolling body faults. Different types of bearing faults exhibit distinct fault characteristic frequencies, which can be understood as low-frequency vibrations produced during the operation of the bearing, where the fault point periodically collides with other non-fault parts of the bearing [14]. These frequencies relate to the bearing’s geometric dimensions, the location of the damage, and the motor’s speed. Since the outer ring of the bearing used in a PMSM is generally stationary while the inner ring rotates with the rotor, the outer ring is more prone to damage [15]. Thus, this study focuses solely on the experimental analysis of localized faults on the outer ring. The calculation formula for the characteristic frequency of outer ring faults is as follows [16]:

(1) $$\begin{array}{c} f_{\mathrm{BPOF}}=N_{B}\left|\frac{f_{s}}{2}\left(1-\frac{D_{b}}{D_{c}}\cos\theta \right)-0\;\right|\\ =\frac{N_{B}f_{s}}{2}\left(1-\frac{D_{b}}{D_{c}}\cos\theta \right) \end{array}$$

where *f_BPOF_* is the characteristic frequency of the outer ring fault, *N_B_* is the number of rolling elements, *f_s_* is the rotor speed frequency, *D_b_* is the diameter of the rolling elements, *D_c_* is the diameter of the cage, and *θ* is the contact angle of the bearing.

According to studies [17], for most bearings with 6 to 12 balls, the outer ring fault characteristic frequency can be approximated as follows:

(2) $$f_{\mathrm{BPOF}}=0.4N_{B}f_{s}$$

It is generally believed that bearing faults lead to the rotational eccentricity of the rolling bearing, leading to periodic variations in the air gap. This variation affects the magnetic flux density in the air gap, thereby causing periodic changes in the motor’s inductance. Consequently, the stator current of the motor is also subject to periodic variations due to the influence of inductance. This ultimately results in additional frequency components *f_bf_* in the stator current, calculated as follows [18]:

(3) $$f_{\mathrm{bf}}=\left|f_{1}\pm mf_{\mathrm{BPOF}}\right|$$

where *f*_1_ is the power supply frequency and *m* = 1, 2, 3…

In the early stages of bearing faults, due to their minor severity and limited extent, the resulting variations in the motor air gap caused by the bearing faults are relatively small. However, because the motor speed remains constant, the frequency of periodic changes in the motor air gap also remains unchanged. Consequently, during the early stages of bearing faults, while the magnitude of changes in the stator current of the motor is small, the frequency of periodic changes remains constant. This results in the appearance of fault characteristic frequencies in the frequency domain plot of the faulty stator current signal, albeit with small frequency amplitudes and a low signal-to-noise ratio. Therefore, appropriate signal processing methods are necessary to enhance the signal-to-noise ratio, thereby improving the accuracy of early-stage bearing fault diagnosis.

### 2.2. Differential Local Mean Decomposition

DLMD is an improvement over traditional LMD. In DLMD, the signal is first differentiated, which does not change the frequency of the original signal but helps preserve the frequencies with higher energy levels while progressively filtering out smaller, spurious interference signals. This process enables the extraction of fault characteristic signals. The specific steps of DLMD are as follows:

1. Perform *k*-th order differentiation of the original signal to obtain *x*^(*k*)^(*t*).
2. Identify all local extremum points *n_i_* of the differentiated signal *x*^(*k*)^(*t*), and calculate the average of all adjacent local extremum points:
  (4) $$m_{i}=\frac{n_{i}+n_{i+1}}{2}$$
  where *n_i_* is the *i*-th local extremum point, *n_i+_*_1_ is the (*i*+1)-th local extremum point, and *m_i_* is the *i*-th mean value. Because the number of *n_i_* characterizes the degree of waveform distortion and the content of impulse components in the *x*^(*k*)^(*t*), the number of *m_i_* can also reflect the degree of waveform distortion and the content of impulse components in the signal. Connect all adjacent mean points *m_i_* with straight lines and apply a moving average method to smooth the resultant local mean function *m*_11_(*t*). This function reflects the overall trend and periodic variations of the signal. Clearly, *m*_11_(*t*) is influenced by the mean points *m_i_*. However, compared to the number of *m_i_*, the values of *m_i_* have a more significant impact on *m*_11_(*t*). If the differences between each mean point are large, then the fluctuation of the local mean function *m*_11_(*t*) will be large; conversely, if the differences are small, the fluctuation will be small. Therefore, the distribution of *m_i_* has an impact on the subsequent signal decomposition process in the form of the local mean function *m*_11_(*t*).
3. Obtain the envelope estimation value.
  (5) $$a_{i}=\frac{\left|n_{i}-n_{i+1}\right|}{2}$$
  Connect all adjacent mean points *a_i_* with straight lines and apply a moving average method to smooth the resultant envelope estimation function *a*_11_(*t*).
4. Subtract the local mean function *m*_11_(*t*) from the signal *x*^(*k*)^(*t*) to isolate it:
  (6) $$h_{11}\left(t\right)=x^{\left(k\right)}\left(t\right)-m_{11}\left(t\right)$$
5. Demodulate *h*_11_(*t*) to obtain *s*_11_(*t*).
  (7) $$s_{11}\left(t\right)=\frac{h_{11}\left(t\right)}{a_{11}\left(t\right)}$$
  Repeat steps 2 to 5 to obtain the envelope estimation function *a*_12_(*t*) of *s*_11_(*t*). If *a*_12_(*t*) does not equal 1, then *s*_11_(*t*) is not a pure frequency-modulated signal. Continue the iteration process until *s*_1*n*_(*t*) is a pure frequency-modulated signal. In practice, to reduce the number of iterations, the following condition can be used as the termination criterion for the iterations:
  (8) $$a_{1n}\left(t\right)\approx 1$$
6. Multiply all the envelope estimation functions generated during the iterative process to obtain the envelope signal.
  (9) $$a_{1}\left(t\right)=a_{11}\left(t\right)a_{12}\left(t\right)\ldots a_{1n}\left(t\right)$$
7. Compute the first-order PF of the signal *x*^(*k*)^(*t*).
  (10) $${\mathrm{PF}}_{1}\left(t\right)=a_{1}\left(t\right)s_{1n}\left(t\right)$$
8. Separate PF_1_(*t*) from *x*^(*k*)^(*t*) to get a new signal *u*_1_(*t*).
  (11) $$u_{1}\left(t\right)=x^{\left(k\right)}\left(t\right)-{\mathrm{PF}}_{1}\left(t\right)$$
  Repeat steps 2 to 8 until *u_r_*(*t*) becomes a monotonic function. From this, *r* PF components can be decomposed, denoted as PF*_j_*^(*k*)^ (*t*), where *j* = 1, 2, …, *r*.
9. Integrate each PF once:
  (12) $$\int {\mathrm{PF}}_{j}^{\left(k\right)}\left(t\right)dt=e_{j}^{\left(k-1\right)}\left(t\right)+e_{j0}^{\left(k-1\right)}$$
  where *e_j_*^(*k*−1)^ (*t*) is the antiderivative of the *j*-th PF; *e_j_*_0_^(*k*−1)^ is the integration constant for the *j*-th PF.
10. Decompose each *e_j_*^(*k*−1)^ (*t*) using steps 2 to 8 for first-order decomposition:
  (13) $$e_{j}^{\left(k-1\right)}\left(t\right)={\mathrm{PF}}_{j}^{\left(k-1\right)}\left(t\right)+v_{j}^{\left(k-1\right)}\left(t\right)$$
  where *v_j_*^(*k*−1)^ (*t*) is the *j*-th residual component.
11. Calculate the total residual component.
  (14) $$v_{0}^{\left(k-1\right)}=\sum_{j=1}^{r}v_{j}^{\left(k-1\right)}\left(t\right)+\sum_{j=1}^{r}e_{j0}^{\left(k-1\right)}$$
12. Repeat steps 9 to 11 until performing the integration *k* times, obtaining the PFs of the original signal *x*_0_ (*t*) after DLMD, denoted as PF*_j_*(*t*), *j* = 1, 2, …, *r*, and the residual components. The signal *x*_0_ (*t*) is reconstructed from the r PFs and the residual component *v*_0_ (*t*), as follows:
  (15) $$x_{0}\left(t\right)=\sum_{j=1}^{r}\mathrm{PF}j\left(t\right)+v_{0}\left(t\right)$$
  (16) $$v_{0}\left(t\right)=u_{r}\left(t\right)+\sum_{i=1}^{k}v_{0}^{\left(i\right)}\left(t\right)$$

**Figure 1:**
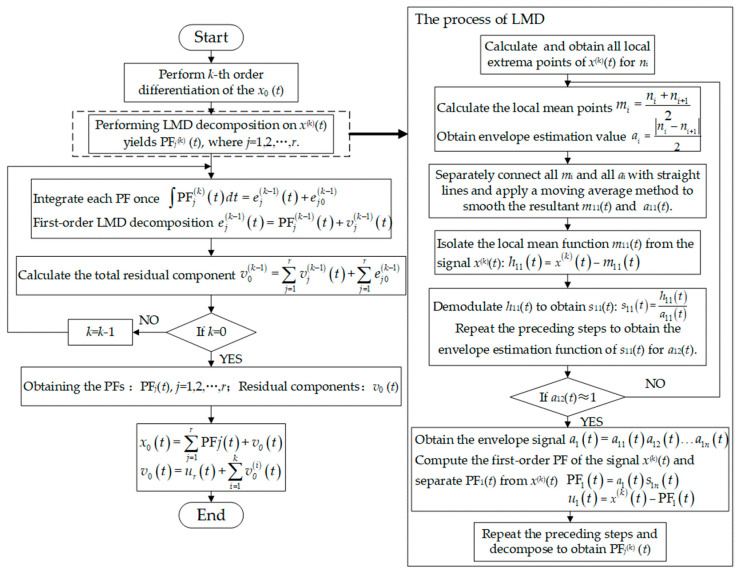
DLMD process.

The specific DLMD process is shown in Figure 1.

### 2.3. Signal-Weighted Fusion

Kurtosis is a dimensionless parameter that is insensitive to bearing speed, size, or load but highly sensitive to impact signals. Therefore, it can be used to judge the strength of signal impacts. The expression for kurtosis is as follows:

(17) $$K=\frac{\frac{1}{n}\sum_{i=1}^{n}{\left(x-\mu \right)}^{4}}{{\left[\frac{1}{n}\sum_{i=1}^{n}{\left(x-\mu \right)}^{2}\right]}^{2}}$$

where *K* is the kurtosis value, *n* is the length of the signal *x*, and *μ* is the mean of the signal *x*.

The kurtosis values of each PF of both the current and vibration signals are calculated. The importance of each PF obtained from decomposition can be measured by its kurtosis value. Therefore, thresholds for filtering PFs can be selected based on kurtosis values. The specific threshold determination will be analyzed in the experimental section below. The weighting coefficients for signal fusion are calculated as follows:

(18) $$\lambda_{Ij}=\frac{K_{\mathrm{Ij}}p_{I}}{\sum_{j=1}^{p_{I}}K_{\mathrm{Ij}}}$$

(19) $$\lambda_{\mathrm{Vj}}=\frac{K_{\mathrm{Vj}}p_{V}}{\sum_{j=1}^{p_{V}}K_{\mathrm{Vj}}}$$

where *λ_Ij_* and *λ_Vj_* are the weighting coefficients for each PF of the current and vibration signals, respectively; *K_Ij_* and *K_Vj_* are the kurtosis values for each PF of the current and vibration signals, respectively; and *p_I_* and *p_V_* are the numbers of PFs selected for fusion from the current and vibration signals, respectively.

Once the weighting coefficients are determined, the current and vibration signals are reconstructed using these weights as follows:

(20) $$X_{I}=\sum_{j=1}^{p_{I}}\lambda_{\mathrm{Ij}}\times PF_{\mathrm{Ij}}$$

(21) $$X_{V}=\sum_{j=1}^{p_{V}}\lambda_{\mathrm{Vj}}\times PF_{\mathrm{Vj}}$$

where *X_I_* and *X_V_* are the weighted reconstructed current and vibration signals, respectively.

Due to the influence of different signal dimensions and units on analysis accuracy, it is necessary to normalize the reconstructed current and vibration signals before fusing them. The normalization process maps the data to a range of 0 to 1 as follows [12]:

(22) $$X_{I}^{\prime }\left(t\right)=\frac{X_{I}\left(t\right)-X_{\mathrm{Imin}}\left(t\right)}{X_{\mathrm{Imax}}\left(t\right)-X_{\mathrm{Imin}}\left(t\right)}$$

(23) $$X_{V}^{\prime }\left(t\right)=\frac{X_{V}\left(t\right)-X_{\mathrm{Vmin}}\left(t\right)}{X_{\mathrm{Vmax}}\left(t\right)-X_{\mathrm{Vmin}}\left(t\right)}$$

After normalizing the reconstructed current and vibration signals, the two signals are fused to obtain a current–vibration fusion signal:

(24) $$X\left(t\right)=X_{I}^{\prime }\left(t\right)+X_{V}^{\prime }\left(t\right)$$

By fusing the current and vibration signals with weighted reconstruction, the signal-to-noise ratio is effectively improved, suppressing noise interference, and thereby highlighting the fault characteristic frequencies.

### 2.4. Hilbert Transform

The Hilbert transform is one of the main methods used to extract the envelope of a signal [19]. Given a continuous-time signal *X*(*t*), its Hilbert transform is defined as follows:

(25) $$\;\hat{X}\;(t)=H(X(t))=\frac{1}{\pi }\int_{-\infty }^{\infty }\frac{X(\tau )}{t-\tau }d\tau =X(t)*\frac{1}{\pi t}$$

where *H*(•) is the Hilbert operator and * denotes convolution.

With *X* (*t*) as the real part and its Hilbert transform as the imaginary part, the complex signal defined as the analytic signal *Z*(*t*) of *X*(*t*) is:

(26) $$Z(t)=X(t)+j\;\hat{X}\;(t)=\alpha (t)e^{j\varphi (t)}$$

The instantaneous amplitude and phase of the analytic signal *Z* (*t*) are then:

(27) $$\alpha (t)=\left|Z(t)\right|=\sqrt{X^{2}(t)+{\hat{X}}^{2}\left(t\right)}$$

(28) $$\varphi (t)=\arctan\left(\frac{\hat{X}\;(t)}{X(t)}\right)$$

According to the analysis in reference [20], during the process of applying Hilbert transformation to the stator fault current signal and taking the envelope, the feature frequency detected can be shifted from the sideband components analyzed above, |*f*_1_ ± *mf_BPOF_*|, to directly detect the outer ring fault frequency, *f_BPOF_*. Therefore, after signal fusion of the current and vibration signals, the existence of the outer ring fault frequency *f_BPOF_* in the fused signal can be directly detected to determine if there is a fault in the bearing of the outer ring.

## 3. Experimental Setup and Procedure

### 3.1. Experimental Setup

**Figure 2:**
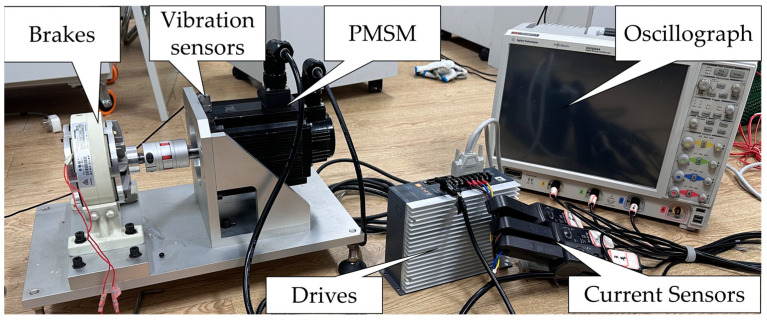
Motor experiment platform.

**Figure 3:**
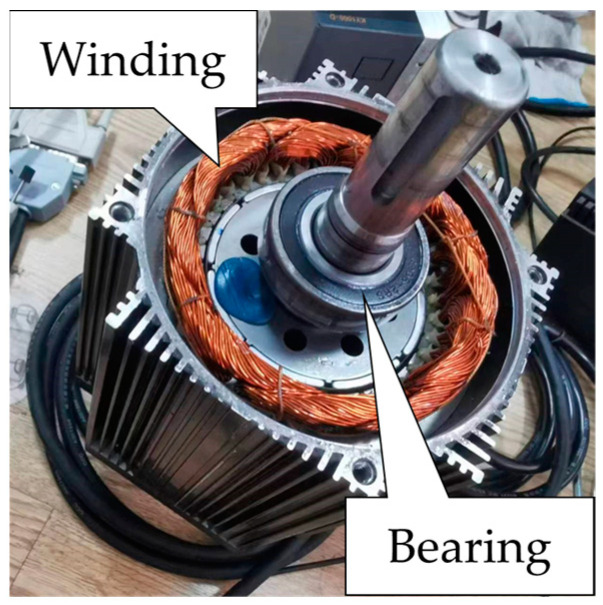
The bearing position within the motor.

The experimental platform used in this study consists of three parts: a power system, a motor drive system, and a data acquisition system. The power system supplies electricity at a frequency of 100 Hz. The motor used in the experiment is an 8-pole, 36-slot PMSM with a rated power of 1.5 kW. The data acquisition system includes a vibration data collector, a current transformer, and an oscilloscope. The motor experimental platform is shown in Figure 2, and the position of the bearing within the motor is shown in Figure 3.

**Figure 4:**
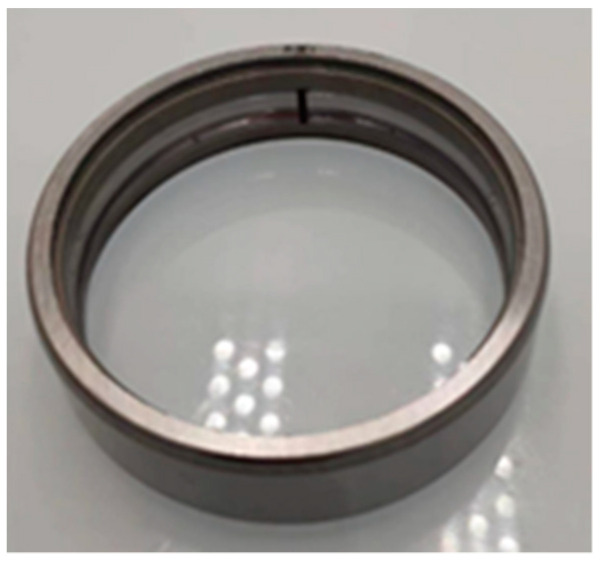
Faulty bearing.

The experiment is conducted with the motor running unloaded. Because the fault extent is relatively small, it is common in research to simulate it using cracks or holes. Drawing from the rolling bearing fault experimental scheme at CWRU [21,22], this study utilizes a rolling bearing with a 0.2 mm deep notch on its outer ring to simulate early-stage faults in the outer ring, as shown in Figure 4. The motor’s speed is stabilized at 1500 rpm, and both stator current and vibration signals are sampled at a frequency of 100,000 Hz with a total of 100,000 sample points each. The sampled data are then processed using Matlab R2021b.

### 3.2. Experimental Procedure

**Figure 5:**
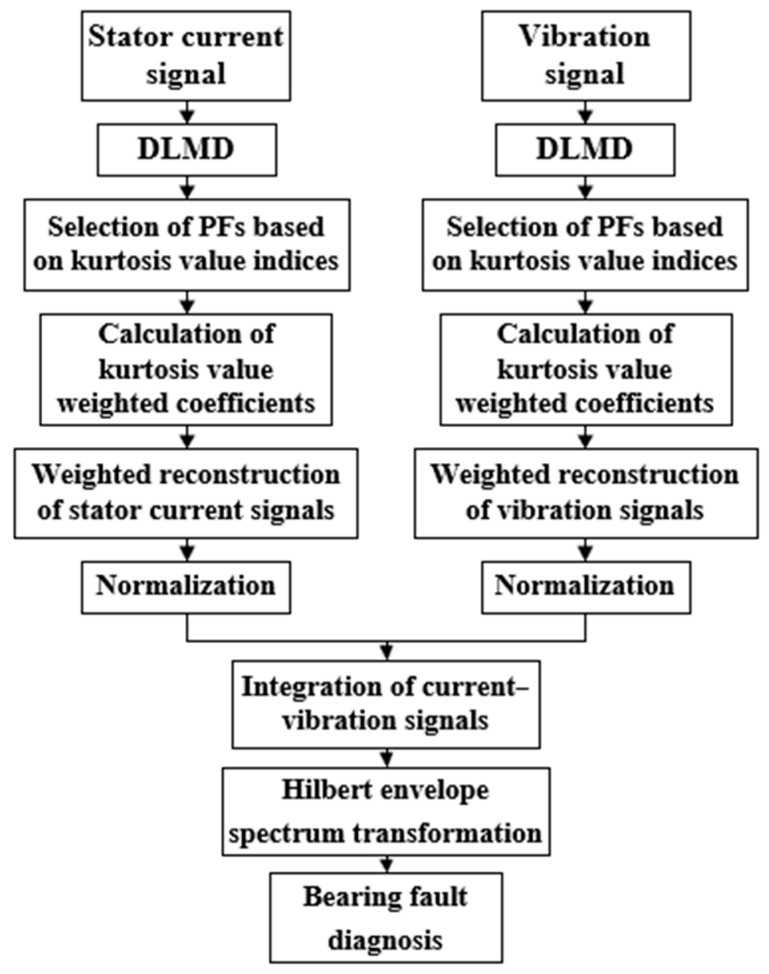
Fault diagnosis process.

The proposed method based on DLMD and current–vibration signal fusion makes full use of the adaptive signal processing characteristics of DLMD, combined with kurtosis weighting to construct a current–vibration fusion signal with pronounced impact features. Finally, a Hilbert envelope spectrum transformation is applied to obtain a clear fault characteristic frequency. The fault diagnosis process is shown in Figure 5.

The specific steps are as follows:

1. Set the predetermined sampling rate on the motor experiment platform and collect the fault stator current and fault vibration signals using vibration sensors and current transformers when the outer ring fault occurs.
2. Perform DLMD on the collected original vibration and stator current signals, setting the number of PFs to 10. To avoid modal aliasing and to minimize the amount of arithmetic, the number of differentiations *k* is set to 3.
3. Calculate the kurtosis values of each PF using Equation (17), then the kurtosis values are used to filter the PFs and calculate the kurtosis weighted coefficients.
4. Perform weighted reconstruction of both the stator current signal and the vibration signal according to the kurtosis weighting coefficients.
5. Normalize both the reconstructed stator current signal and the vibration signal, then fuse them to obtain the current–vibration fusion signal.
6. Analyze the current–vibration fusion signal using the Hilbert envelope spectrum to obtain the fault diagnosis results.

## 4. Experimental Results and Analysis

**Figure 6:**
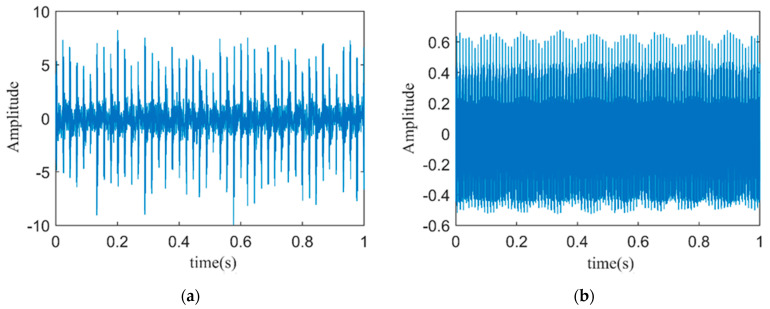
(**a**) Time–domain plot of fault vibration signals; (**b**) Time–domain plot of fault stator current signals.

To verify the effectiveness of the proposed signal processing method, fault vibration signals and stator current signals from a PMSM with an outer ring fault are sampled under no-load experimental conditions. The vibration time-domain signal and the stator current time-domain signal are shown in Figure 6.

The theoretical calculations based on motor parameters and operational conditions yield an outer ring fault characteristic frequency *f_BPOF_* of 89.3 Hz; the fundamental frequency *f*_1_ of the power system is 100 Hz, and the motor’s rotation frequency *f_s_* is 25 Hz.

### 4.1. Vibration Signal-Weighted Reconstruction

**Figure 7:**
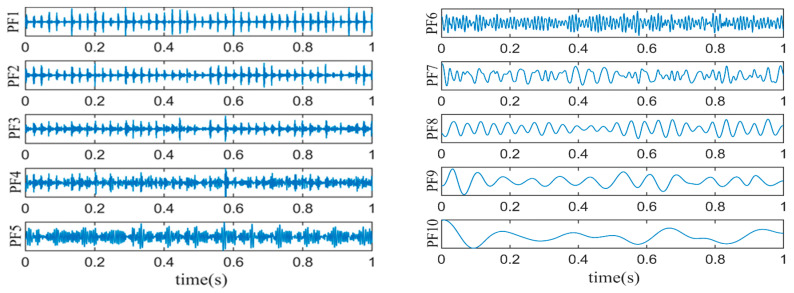
DLMD results of fault vibration signals.

DLMD is performed on the fault vibration signal, as high-order PFs contain less fault information; thus, the number of PFs is set to 10. The results of the DLMD are shown in Figure 7.

**Figure 8:**
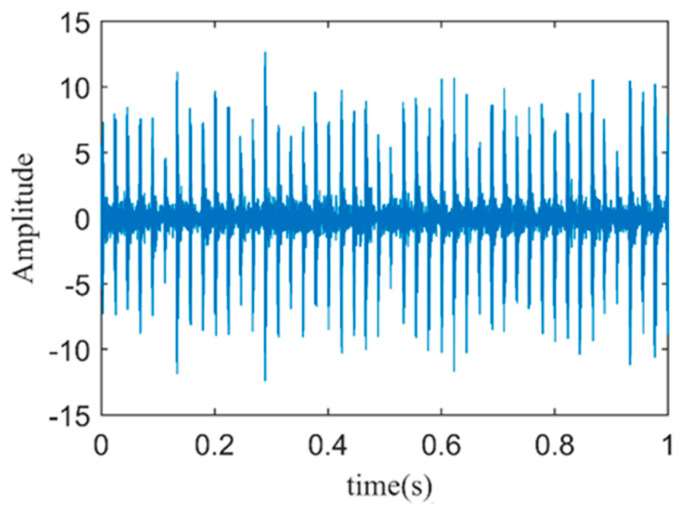
Time–domain plot of vibration–weighted reconstructed signal.

Because the kurtosis value of a normal distribution signal is three, in order to preserve more fault impact features, PFs with kurtosis values greater than three are selected for signal-weighted reconstruction [23]. According to Equations (17)–(19), the kurtosis values and weighting coefficients for each PF are calculated, as shown in Table 1. Components with a kurtosis value of less than three are filtered out, and thus the weighting coefficients are set to zero. The reconstructed signal is shown in Figure 8.

**Figure 9:**
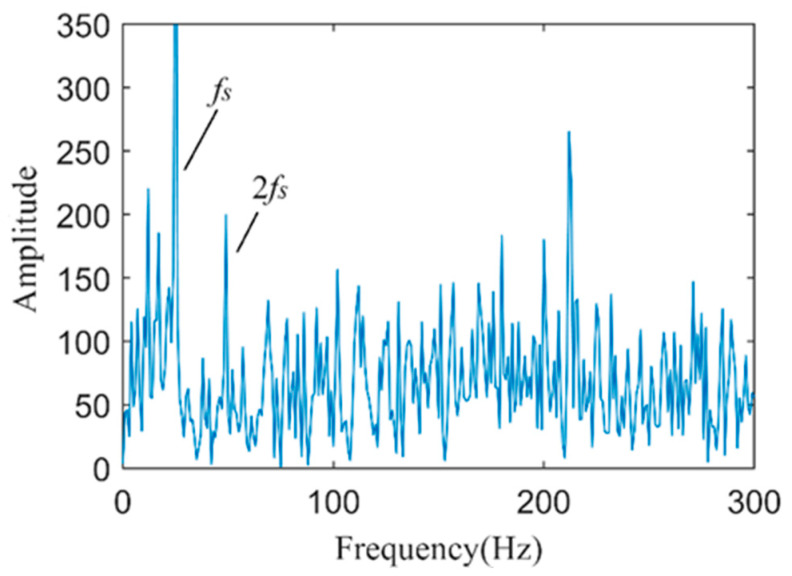
The spectrum of the envelope of the vibration-weighted reconstructed signal.

Figure 9 shows the Hilbert envelope spectrum of the vibration-weighted reconstructed signal. From the figure, the motor rotation frequency *f_s_* and its second harmonic are quite apparent, but the environmental background noise is substantial, making it difficult to clearly identify the fault characteristic frequency *f_BPOF_* in the envelope spectrum. It is evident that directly applying weighted reconstruction and Hilbert envelope analysis to the fault vibration signal cannot effectively or accurately diagnose early-stage outer ring faults in bearings.

### 4.2. Stator Current Signal-Weighted Reconstruction

**Figure 10:**
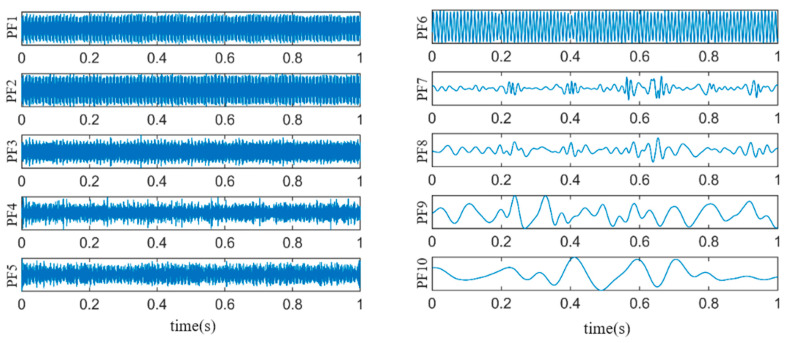
DLMD results of fault stator current signals.

The DLMD is applied to the stator current signal, and the PFs obtained are shown in Figure 10. Just like with the vibration signals, kurtosis values and weighting coefficients for each PF of the stator current signal are calculated, as shown in Table 2.

Based on the analysis, the PFs of the fault stator current signal are mostly distorted sinusoidal signals, containing fewer impact characteristics; thus, kurtosis values are generally lower. Therefore, its filtering threshold needs to be less than three. Moreover, from the calculation results, the kurtosis values of most PFs are greater than two. Thus, a filtering threshold between two and three can be selected.

**Figure 11:**
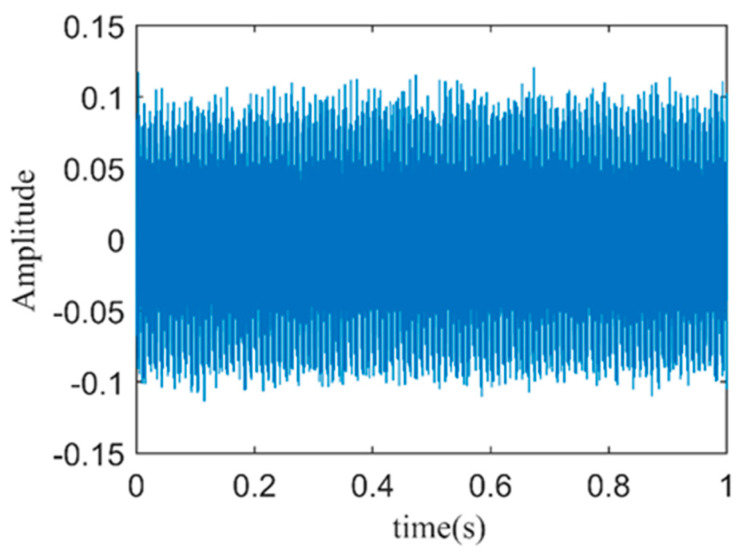
Time–domain plot of weighted reconstructed stator current signal.

Next, by analyzing the envelope spectrum of the weighted reconstructed stator current signal under different filtering thresholds, in terms of the fault characteristic frequency amplitude and the average amplitude of background noise, the optimal filtering threshold can be determined. The calculation method for the average amplitude of background noise involves averaging the amplitudes of all frequencies after filtering out the power system frequency *kf*_1_, the motor’s rotation frequency *kf_s_*, and the fault characteristic frequency *f_BPOF_*. The analysis results are presented in Table 3. It can be observed that when the filtering threshold is set to two, the signal-to-noise ratio of the reconstructed signal envelope spectrum is maximized. Therefore, PFs with kurtosis values greater than two are chosen for the weighted reconstruction of the faulty stator current signal. The reconstructed signal is shown in Figure 11.

**Figure 12:**
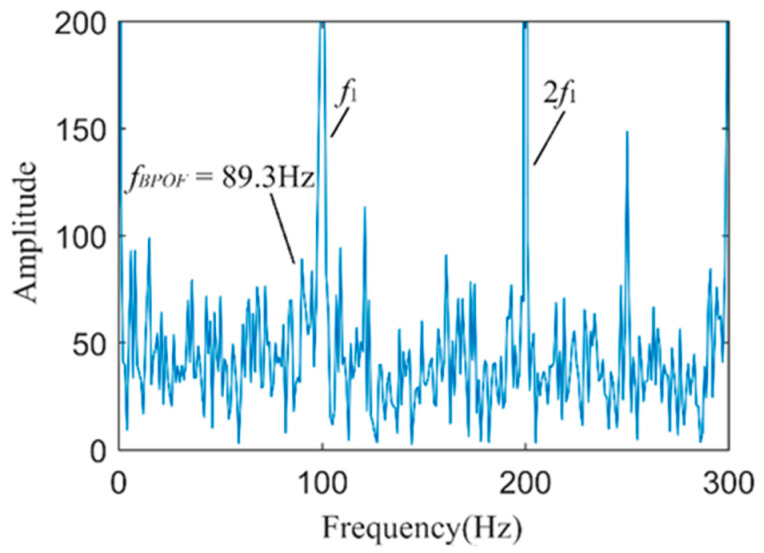
The spectrum of the envelope of the weighted reconstructed stator current signal.

As shown in Figure 12, the Hilbert envelope spectrum of the weighted reconstructed stator current signal is presented. From the figure, it is evident that the fundamental frequency *f*_1_ and its harmonics are clearly visible in the envelope spectrum. Additionally, the fault characteristic frequency *f_BPOF_* can be identified in the envelope spectrum. However, its magnitude is close to the surrounding noise and harmonic components, which may severely affect the accuracy of bearing fault diagnosis. This indicates that directly reconstructing the faulty stator current signal with weighting and obtaining its Hilbert envelope spectrum may not effectively and accurately diagnose early-stage faults in the outer ring of the bearing.

### 4.3. Direct Fusion Analysis of Current–Vibration Signal

**Figure 13:**
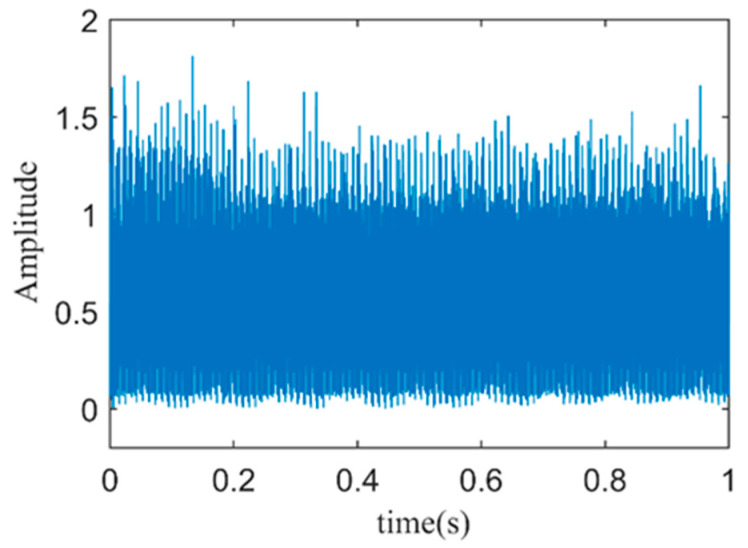
Time-domain plot of directly fused current–vibration signal.

To verify the effectiveness of current–vibration signal fusion in bearing fault diagnosis, the fault vibration signal and fault stator current signal are directly normalized using the Equations (22)–(24), and then fused. The time-domain plot of the directly fused current–vibration signal is shown in Figure 13.

**Figure 14:**
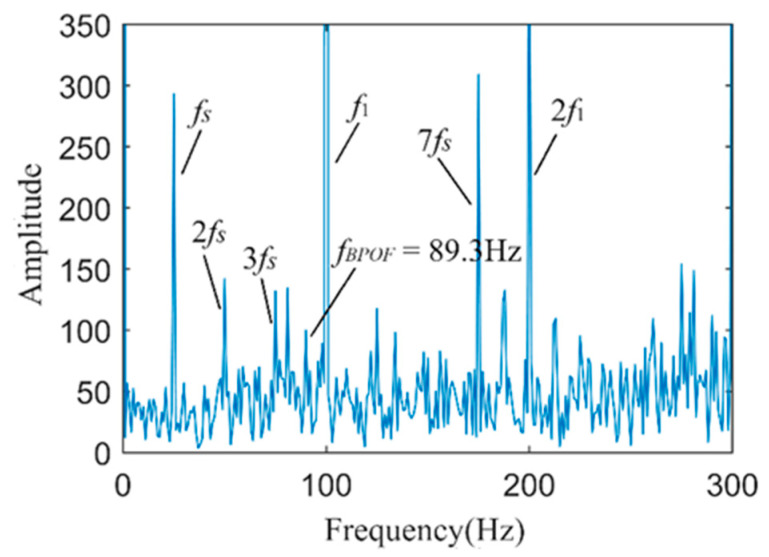
The spectrum of the envelope of the current–vibration directly fused signal.

The Hilbert envelope spectrum of the directly fused current–vibration signal is shown in Figure 14. As analyzed earlier, after envelope spectrum analysis of the fused signal, it is possible to directly detect the outer ring fault characteristic frequency *f_BPOF_* to determine if a fault has occurred in the outer ring of the motor bearing. The envelope spectrum clearly shows the motor rotation frequency *f_s_*, the fundamental frequency of the power system *f*_1_, and their multiples. Although the fault characteristic frequency *f_BPOF_* can be identified more clearly, its amplitude is significantly smaller than that of the motor rotation frequency and the power system fundamental frequency, which may cause interference in fault diagnosis.

### 4.4. Weighted Fusion Analysis of Current–Vibration Signal

**Figure 15:**
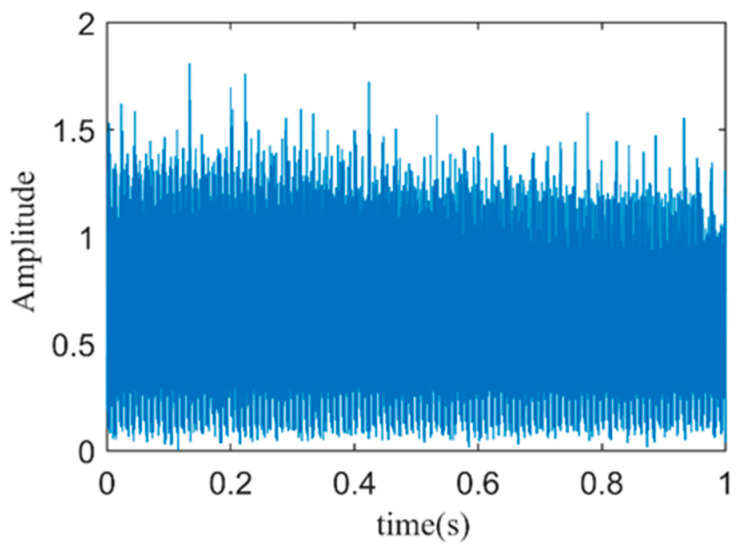
Time-domain plot of current–vibration-weighted fusion signal.

Following the experimental procedure described in Section 3.2 and the experimental results analyzed earlier, the weighted reconstructed stator current signal and vibration signal are normalized and then fused. The time-domain plot of the current–vibration-weighted fusion signal is shown in Figure 15.

**Figure 16:**
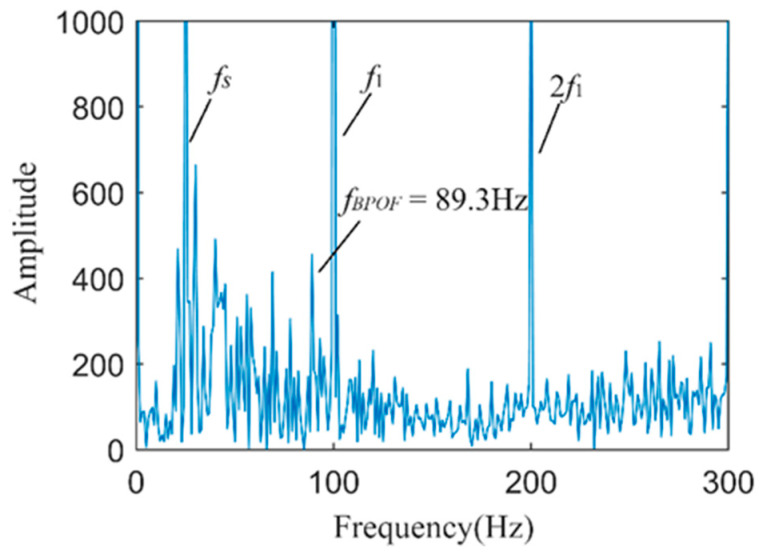
The spectrum of the envelope of the current–vibration-weighted fused signal.

The Hilbert envelope spectrum of the current–vibration-weighted fusion signal is shown in Figure 16. The spectrum clearly shows the motor rotation frequency *f_s_*, the fundamental frequency of the power system *f*_1_, and their multiples. Additionally, the fault characteristic frequency *f_BPOF_* can be distinctly identified, with its amplitude being significantly greater than the surrounding noise harmonics, enabling effective distinction between noise harmonics and inherent motor frequencies. This indicates that the weighted fusion of the stator current signal and vibration signal followed by Hilbert envelope spectrum analysis can significantly improve the signal-to-noise ratio of the fault characteristic signal, effectively suppress environmental noise and the motor’s inherent frequency interference, and enhance the effectiveness and accuracy of early fault diagnosis in the outer rings of the motor bearing.

## 5. Discussion

The early-stage fault diagnosis method proposed in this paper can significantly improve the signal-to-noise ratio of fault characteristic signals, effectively suppress modal aliasing during signal decomposition, and reduce the interference of environmental noise and the motor’s inherent frequencies on fault diagnosis. This enhances the accuracy of identifying early weak fault signals in bearings. However, there is still the possibility of further optimizing and refining this method, which can be considered from the following three aspects:

1. In this paper, DLMD is chosen as the signal decomposition method, which, compared to EMD, mitigates to some extent mode mixing and endpoint effects, enabling a more accurate reflection of all the characteristic information of the original signal. However, there are still issues such as the inability to determine the differential order *k* reasonably and the inability to highlight impulse components. Therefore, further optimization of the determination criteria for the differential order *k* can be achieved through methods such as Hilbert demodulation [24], among others.
2. This paper employs kurtosis as the criterion for filtering and weighting PFs because kurtosis is sensitive to impulse components in signals and can effectively assess the presence of fault characteristic information in each PF. However, kurtosis, as a fourth-order statistical moment, is also highly sensitive to extreme values, and random impulses generated by signal extremes can significantly affect the calculation of signal kurtosis. Therefore, integrating parameters such as correlation coefficients and the Sparsity of Envelope Spectrum [25,26] can further optimize the filtering and weighting reconstruction of PFs, thereby enhancing the signal-to-noise ratio and suppressing the interference of noise on fault diagnosis.
3. This paper presents experimental and analytical investigations on the diagnosis of localized outer ring faults in motor bearings using vibration signals and stator current signals. Vibration signals and stator current signals are highly sensitive to bearing faults and can effectively reflect the early weak signal characteristics of bearing failures. Additionally, other motor signals such as acoustic signals and temperature rise signals also contain information about bearing faults and are utilized in diagnosing motor bearing faults. Therefore, integrating a wider range of motor signals in bearing fault diagnosis can lead to the development of more comprehensive and accurate diagnostic models.
4. This study simulates localized outer ring faults in motor bearings using cracks and does not include experimental analysis of wear-type faults or faults in other bearing locations. However, faults can also occur in other parts of the motor bearing, such as the inner ring and rolling elements. While wear-type faults may not significantly impact motor operation in their early stages, they can still affect normal motor operation as the faults accumulate over time. Therefore, further research and analysis of different locations and types of bearing faults can enhance the comprehensiveness and generalizability of diagnostic methods.
5. The early-stage fault diagnosis method proposed in this paper utilizes the presence of fault characteristic frequencies in the weighted fusion of a signal envelope spectrum as the diagnostic basis for bearing fault diagnosis. During bearing faults, the motor rotor undergoes periodic vibration eccentricity, resulting in periodic variations in the air gap and hence the appearance of the motor rotation frequency and its harmonics in the stator current spectrum. Additionally, the stator current spectrum contains a power supply frequency and its harmonics as well as significant environmental noise. Although methods such as signal filtering and weighted reconstruction effectively improve the signal-to-noise ratio, the presence of this environmental noise and the motor’s inherent frequencies still severely interferes with the direct identification of fault characteristic frequencies, making it difficult to accurately determine whether the motor bearing is faulty. Therefore, machine learning can be employed to optimize the early-stage bearing fault diagnosis method proposed in this paper [27,28]. By constructing a fault diagnosis model and training it with a large amount of weighted fusion of a signal envelope spectrum from early-stage bearing fault scenarios, the model can adaptively identify bearing fault characteristic frequencies, eliminating the subjective limitations of manual fault characteristic frequency identification and improving diagnosis accuracy. Moreover, the use of machine learning enables the online diagnosis of bearing faults. During motor operation, vibration signals and stator current signals can be directly collected and analyzed, and the resulting envelope spectrum can be input into the trained fault diagnosis model to determine whether the motor bearing is faulty.

## 6. Conclusions

The stator current signal and vibration signal of PMSM contain bearing fault information, which manifests in the spectrum as specific fault characteristic frequencies. However, due to the low-frequency amplitude and signal-to-noise ratio of early-stage fault signals, direct envelope spectrum analysis of either stator current signals or vibration signals, or simple fusion of the two followed by envelope spectrum analysis, cannot effectively and accurately diagnose early-stage faults in the outer ring of permanent magnet synchronous motor bearings. Therefore, this paper proposes a bearing fault diagnosis method based on DLMD and current–vibration signal fusion. This method, through DLMD and signal-weighted fusion, significantly enhances the signal-to-noise ratio of fault characteristic signals, effectively suppresses mode mixing, and reduces environmental noise and the motor’s inherent frequency interference, improving spectral analysis quality and facilitating the capture of fault characteristic frequencies. The effectiveness of the proposed method is validated through experimental analysis under no-load conditions of a faulted motor.

Due to limitations in experimental conditions and other factors, this study focuses solely on the experimental analysis of localized outer ring faults in bearings. Additionally, the proposed fault diagnosis method requires manual identification of fault characteristic signals, which introduces subjective limitations and hinders the realization of online real-time bearing fault diagnosis. Therefore, future research should continue to investigate faults in different locations and of various types to enhance the comprehensiveness and generalizability of diagnostic methods. Furthermore, integrating machine learning, cloud computing, and adaptive diagnostic technologies will be necessary to achieve online real-time diagnosis of motor bearing faults, thereby further ensuring the safe operation of motors.

## Figures and Tables

**Table 1:** Kurtosis values and weighting coefficients of each order PF of fault vibration signals.

Product Function	Kurtosis Values	Weighting Coefficients
PF 1	7.8533	1.6635
PF 2	4.5896	0.9722
PF 3	4.0154	0.8505
PF 4	3.5977	0.7621
PF 5	2.9875	0
PF 6	2.9004	0
PF 7	3.5489	0.7517
PF 8	2.8752	0
PF 9	2.0157	0
PF 10	1.7563	0

**Table 2:** Kurtosis values and weighting coefficients of each order PF of stator current signals.

Product Functions	Kurtosis Values	Weighting Coefficients
PF 1	1.8515	0
PF 2	2.4631	0.9395
PF 3	2.2483	0.8576
PF 4	3.0875	1.1777
PF 5	2.8132	1.0731
PF 6	1.7963	0
PF 7	2.5056	0.9557
PF 8	2.6124	0.9965
PF 9	1.9418	0
PF 10	1.8565	0

**Table 3:** The amplitude of the fault characteristic frequency and background noise.

Threshold	Fault Characteristic Frequency	Background Noise	Signal-to-Noise Ratio
2	93.5	52.3	1.788
2.4	97.0	55.8	1.738
2.8	71.4	59.4	1.202
3	47.7	60.1	0.794

## Data Availability

Data are contained within the article.
